# Supplementary material for: Enhancement of Antioxidant and Anti-Glycation Properties of Beeswax Alcohol in Reconstituted High-Density Lipoprotein: Safeguarding against Carboxymethyllysine Toxicity in Zebrafish
Source: Antioxidants (Basel). 2023 Dec 14;12(12):2116. doi: 10.3390/antiox12122116 (PMC10740997; doi:10.3390/antiox12122116)
Supplement: Supplementary file 1 [file antioxidants-12-02116-s001.zip › antioxidants-2728276-supplementary.pdf]

Supplementary material

Supplementary Figure S1. Certificate of analysis (COA) for beeswax alcohol (BWA) from inspection of imported food, issued by Korean FDA

Print date:October 5, 2022

Inspection results for imported food, etc.

Income declaration person (Import shipper)	Mutual	Reidel Korea Co., Ltd.	representative	[illegible]
	address	Room 205, 2nd floor, 15-10 Gangnam-daero 39-gil, Seocho-gu, Seoul (Seocho-dong, Halla Vivaldi Studio)		
Registration Number		202200608546	Date of receipt	September 26, 2022
product name				
Type of inspection				
Requesting agency				
Request number				
Inspection agency				
Judgment				

정보공개용 자료

Test/Inspection Item name				
Appearance				010-1) Sample
Octacosanol				010-1) Sample
1-triacotanol	① 1-tetracosanol(C24): 60~150mg/g ② 1-hexacosanol (C26):	292.25	fitness	Beeswax alcohol (No. 2010-1) sample [illegible]
1-dotriacotanol	① 1-tetracosanol(C24): 60~150mg/g ② 1-hexacosanol (C26):	222.97	fitness	Beeswax alcohol (No. 2010-1) sample [illegible]
1-tetracosanol, 1-hexacosanol, 1-octacosanol, 1-triacotanol, 1-dotriacotanol,	① 1-tetracosanol(C24): 60~150mg/g ② 1-hexacosanol (C26):	859.79	fitness	Beeswax alcohol (No. 2010-1) sample [illegible]
lead	0.5 or less	0.1	fitness	Beeswax alcohol (No. 2010-1) sample [illegible]
total arsenic	0.1 or less	Non-detected	fitness	Beeswax alcohol (No. 2010-1) sample [illegible]
total mercury	0.5 or less	Non-detected	fitness	Beeswax alcohol (No. 2010-1) sample [illegible]
cadmium	0.5 or less	Non-detected	fitness	Beeswax alcohol (No. 2010-1) sample [illegible]
acetone	30.0 or less	2.73	fitness	Beeswax alcohol (No. 2010-1) sample [illegible]
hexane	5.0 or less	0.44	fitness	Beeswax alcohol (No. 2010-1) sample [illegible]
1-tetracosanol	① 1-tetracosanol(C24): 60~150mg/g ② 1-hexacosanol (C26):	63.67	fitness	Beeswax alcohol (No. 2010-1) sample [illegible]
1-Hexacosanol	① 1-tetracosanol(C24): 60~150mg/g ② 1-hexacosanol (C26):	114.28	fitness	Beeswax alcohol (No. 2010-1) sample [illegible]

Test/Inspection Item name	standard	result	Judgment	note
coliforms	voice	voice	fitness	Beeswax alcohol (No. 2010-1) sample [illegible]
fatty alcohol	① 1-tetracosanol(C24): 60~150mg/g ② 1-hexacosanol (C26):	30.62	fitness	Beeswax alcohol (No. 2010-1) sample [illegible]

These test results are limited to the samples requested for testing, and the test results must not be used for false, exaggerated labeling, advertising, or slander purposes. If you wish to use them for labeling or advertising, the entire test results must be truthfully displayed. It must be advertised.

September 30, 2022

Director of the Korea Food Science Research Institute, an affiliate of the Korea Food Industry Association

Print date:October 5, 2022

Inspection results for imported food, etc.

[illegible] (Import shipper)	Mutual	Reidel Korea Co., Ltd.	Representative	[illegible]
	address	Room 205, 2nd floor, 15-10 Gangnam-daero 39-gil, Seocho-gu, Seoul (Seocho-dong, Halla Vivaldi Studio)		
Registration Number		202200608546	Date of receipt	September 26, 2022
product name				
Type of inspection				
Requesting agency				
Request number				
Inspection agency				
Judgment				

정보공개용 자료

Test/Inspection Item name				
Desulfonylchlorosilide				
glimepiride				
glibenclamide	Not detected	Not detected	fitness	Sample (1)
glee closet	Not detected	Not detected	fitness	Sample (1)
glyphigit	Not detected	Not detected	fitness	Sample (1)
norneobardenafil	Not detected	Not detected	fitness	Sample (1)
Norneosildenafil	Not detected	Not detected	fitness	Sample (1)
Nitrodenafil	Not detected	Not detected	fitness	Sample (1)
demethyl reddenafil	Not detected	Not detected	fitness	Sample (1)
desulfovadenafil	Not detected	Not detected	fitness	Sample (1)
Desmethylsibutramine	Not detected	Not detected	fitness	Sample (1)
Didesmethylsibutramine	Not detected	Not detected	fitness	Sample (1)
Dimethylsildenafil	Not detected	Not detected	fitness	Sample (1)

Test/Inspection Item name	standard	result	judgment	note
Dimethylthiosildenafil	Not detected	Not detected	fitness	Sample (1)
Dichlorodenafil	Not detected	Not detected	fitness	Sample (1)
Levothyroxine	Not detected	Not detected	fitness	Sample (1)
Liocironine	Not detected	Not detected	fitness	Sample (1)
Mirodenafil	Not detected	Not detected	fitness	Sample (1)
vardenafil	Not detected	Not detected	fitness	Sample (1)
Benzylsildenafil	Not detected	Not detected	fitness	Sample (1)
pseudovardenafil	Not detected	Not detected	fitness	Sample (1)
Sibutramine	Not detected	Not detected	fitness	Sample (1)
Cinnamildenafil	Not detected	Not detected	fitness	Sample (1)
Sildenafil	Not detected	Not detected	fitness	Sample (1)
Cyclopentinafil	Not detected	Not detected	fitness	Sample (1)
Aminotadalafil	Not detected	Not detected	fitness	Sample (1)
Acetylvardenafil	Not detected	Not detected	fitness	Sample (1)
ephedrine	Not detected	Not detected	fitness	Sample (1)
orlistat	Not detected	Not detected	fitness	Sample (1)
Oxohongdenafil	Not detected	Not detected	fitness	Sample (1)
octylnortadalafil	Not detected	Not detected	fitness	Sample (1)
Udenafil	Not detected	Not detected	fitness	Sample (1)
xanthoanthraphyll	Not detected	Not detected	fitness	Sample (1)
Thiosildenafil	Not detected	Not detected	fitness	Sample (1)
Thioquina Piperipil	Not detected	Not detected	fitness	Sample (1)
Chlohomosildenafil	Not detected	Not detected	fitness	Sample (1)
Carbodenafil	Not detected	Not detected	fitness	Sample (1)

Test/Inspection Item name	standard	result	Judgment	note
chlorodenafil	Not detected	Not detected	fitness	Sample (1)
Chloropretadalafil	Not detected	Not detected	fitness	Sample (1)
tadalafil	Not detected	Not detected	fitness	Sample (1)
Fenfluramine	Not detected	Not detected	fitness	Sample (1)
piperidino reddenafil	Not detected	Not detected	fitness	Sample (1)
Hydroxyvardenafil	Not detected	Not detected	fitness	Sample (1)
Hydroxythiohomosildenafil	Not detected	Not detected	fitness	Sample (1)
Hydroxychlorodenafil	Not detected	Not detected	fitness	Sample (1)
Hydroxyhomosildenafil	Not detected	Not detected	fitness	Sample (1)
Hydroxydenafil	Not detected	Not detected	fitness	Sample (1)
homosildenafil	Not detected	Not detected	fitness	Sample (1)
Hongdenafil	Not detected	Not detected	fitness	Sample (1)
fluoxetine	Not detected	Not detected	fitness	Sample (1)
Sennoside	Not detected	Not detected	fitness	Sample (1)
Yohimbine	Not detected	Not detected	fitness	Sample (1)
Phenolphthalein	Not detected	Not detected	fitness	Sample (1)
Ikarin	Not detected	Not detected	fitness	Sample (1)
N-nitrosofenfluramine	Not detected	Not detected	fitness	Sample (1)
Desmethylpiperazinylpropoxyslide Nafil	Not detected	Not detected	fitness	Sample (1)
Acetaminotadalafil	Not detected	Not detected	fitness	Sample (1)
Demethyltadalafil	Not detected	Not detected	fitness	Sample (1)
avanafil	Not detected	Not detected	fitness	Sample (1)
Methylhydroxyhomosildenafil	Not detected	Not detected	fitness	Sample (1)
Propoxyphenylthiosildenafil	Not detected	Not detected	fitness	Sample (1)

Test/Inspection Item name	standard	result	Judgment	note
Propoxyphenylthioaylldenafil	Not detected	Not detected	fitness	Sample (1)
Propoxyphenylthiohydroxyhomo Sildenafil	Not detected	Not detected	fitness	Sample (1)
Propoxyphenylthiohomosildenafil	Not detected	Not detected	fitness	Sample (1)
Homotadalafil	Not detected	Not detected	fitness	Sample (1)
Chloroxybutramine	Not detected	Not detected	fitness	Sample (1)
cis-cyclopentyltadalafil	Not detected	Not detected	fitness	Sample (1)
trans-cyclopentyltadalafil	Not detected	Not detected	fitness	Sample (1)
Isopropyl Nortadalafil	Not detected	Not detected	fitness	Sample (1)
Descarbonsildenafil	Not detected	Not detected	fitness	Sample (1)
Cascaroside	Not detected	Not detected	fitness	Sample (1)
Dichlopropyl Carbodenafil	Not detected	Not detected	fitness	Sample (1)
Chloroxyphentramine	Not detected	Not detected	fitness	Sample (1)
Acetylic acid	Not detected	Not detected	fitness	Sample (1)
Gendenafil	Not detected	Not detected	fitness	Sample (1)
Imidazogatriazinone	Not detected	Not detected	fitness	Sample (1)

These test results are limited to the samples requested for testing, and the test results must not be used for false, exaggerated labeling, advertising, or slander purposes. If you wish to use them for labeling or advertising, the entire test results must be truthfully displayed. It must be advertised.

September 29, 2022

Seoul Regional Food and Drug Safety Administration

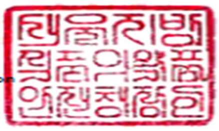

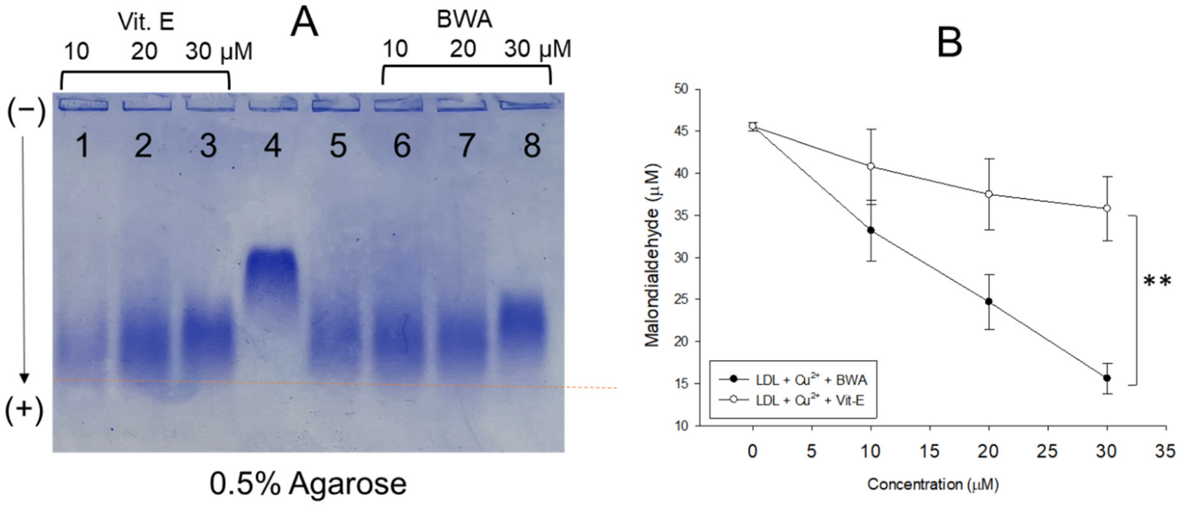

Supplementary Figure S2. Effect of beeswax alcohol (BWA) on $CuSO_4$ induced oxidation of LDL.

A. Electrophoretic mobility of apo-B fraction of LDL. Lane 1, 2, 3, represent LDL+$CuSO_4$ treated with 10, 20, 30, μM vitamin E. Lane 4 represents Native LDL, Lane 5 represents LDL+$CuSO_4$ (Ox LDL). Lane 6, 7, 8, represent LDL+$CuSO_4$ treated with 10, 20, 30, μM BWA. Electrophoresis was performed on 0.5% agarose gel using Tri-EDTA buffer (pH 8.0) at a constant voltage (50 V). Red-dotted line indicates the similar electromobility of oxidized LDL (lane 5) and Vit-E treated ox-LDL (lane 1–3).

B. Quantification of malondialdehyde in the LDL by thiobarbituric acid reactive substances (TBARS) assay. **, $p < 0.01$

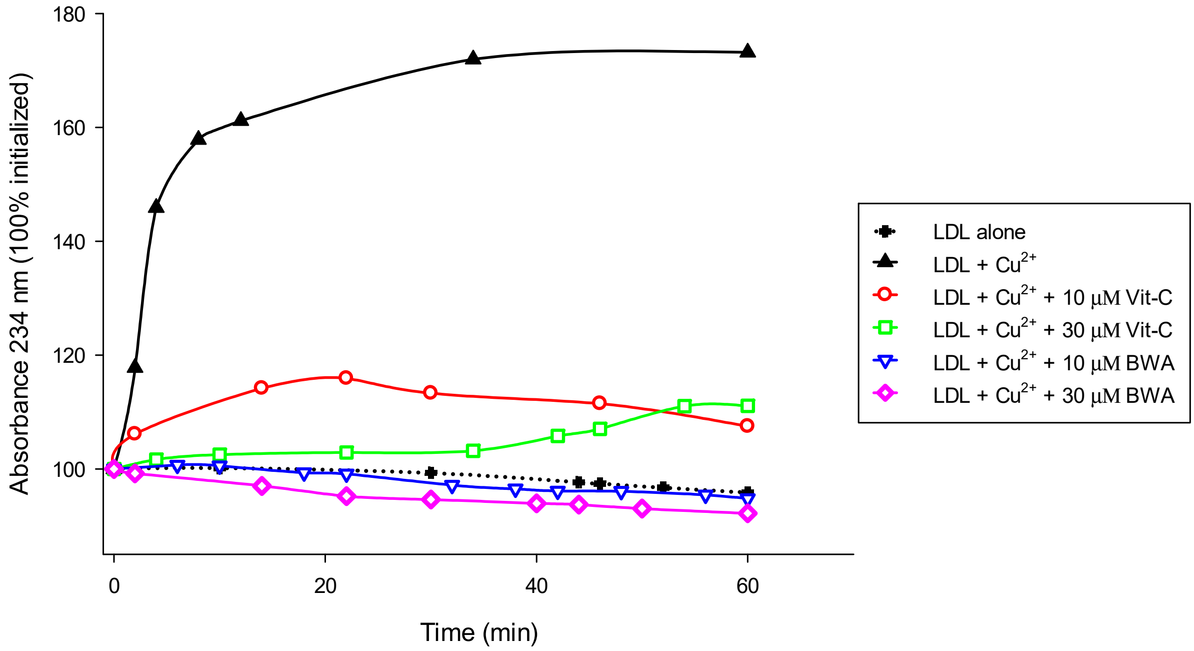

Supplementary Figure S3. Continuous monitoring of conjugated diene detection assay with either beeswax alcohol (BWA) or vitamin C (Vit C) under LDL oxidation by cupric ion treatment.
